# A patient with heart failure and coexistence of constrictive pericarditis and Graves’ disease: a case report

**DOI:** 10.1093/ehjcr/ytaf369

**Published:** 2025-08-04

**Authors:** Yuki Nakata, Eiji Shibahashi, Yuji Iwanami, Hajime Kuroda, Hiroyuki Arashi

**Affiliations:** Department of Cardiology, Tokyo Women’s Medical University Adachi Medical Center, 4-33-1, Kohoku, Adachi-ku, Tokyo 123-8558, Japan; Department of Cardiology, Tokyo Women’s Medical University Adachi Medical Center, 4-33-1, Kohoku, Adachi-ku, Tokyo 123-8558, Japan; Department of Cardiology, Tokyo Women’s Medical University, 8-1, Kawada-cho, Shinjuku-ku, Tokyo 162-8666, Japan; Department of Cardiology, Tokyo Women’s Medical University Adachi Medical Center, 4-33-1, Kohoku, Adachi-ku, Tokyo 123-8558, Japan; Department of Diagnostic Pathology, Tokyo Women’s Medical University Adachi Medical Center, 4-33-1, Kohoku, Adachi-ku, Tokyo 123-8558, Japan; Department of Cardiology, Tokyo Women’s Medical University Adachi Medical Center, 4-33-1, Kohoku, Adachi-ku, Tokyo 123-8558, Japan

**Keywords:** Constrictive pericarditis, Hyperthyroidism, Graves’ disease, Heart failure, Case report

## Abstract

**Background:**

Thyroid disease is associated with pericarditis. There are several case reports on patients with hyperthyroidism and hypothyroidism complicated by acute pericarditis. However, constrictive pericarditis is a rare complication, particularly in hyperthyroidism, and, to the best of our knowledge, it has not been previously reported.

**Case summary:**

A 47-year-old female with no past medical, surgical, or radiotherapy history was admitted to our hospital with a diagnosis of congestive heart failure. Although she was treated for heart failure and hyperthyroidism, the right-sided heart failure did not improve. Right heart catheterization revealed elevated mean right atrial pressure and pulmonary artery wedge pressure. The pressure curve of the right ventricle showed a dip and plateau pattern. The end-diastolic pressure in the left and right ventricles was nearly equal. The cause of heart failure was thought to be constrictive pericarditis, and a pericardiectomy was performed. After surgery, the symptoms and findings of heart failure improved dramatically.

**Discussion:**

We encountered a rare case of heart failure complicated by both constrictive pericarditis and Graves’ disease. Patients with constrictive pericarditis often lack specific laboratory or imaging findings, which can make diagnosis challenging. When treating patients with treatment-resistant right-sided heart failure, it is important to consider constrictive pericarditis and to evaluate haemodynamic evaluation through catheterization.

Learning points

- This report highlights a rare case of heart failure complicated by both constrictive pericarditis and hyperthyroidism.
- In cases of treatment-resistant right-sided heart failure, constrictive pericarditis should be considered in the differential diagnosis, and haemodynamic assessment using cardiac catheterization should be recommended.

## Introduction

Numerous studies have reported an association between thyroid and epicardial diseases.^1^ Thyroid disease is associated with pericarditis, and there have been several case reports of both hyperthyroidism and hypothyroidism complicated by acute pericarditis.^1,2^ However, there are very few reports of constrictive pericarditis as a complication in patients with thyroid disease. In particular, reports of constrictive pericarditis occurring in association with hyperthyroidism are rare, and, to the best of our knowledge, have not been previously described.^1,3^ Graves’ disease is an autoimmune thyroid disorder characterized by excessive thyroid hormone production. In this report, we present a patient with heart failure characterized by the coexistence of thyrotoxicosis and constrictive pericarditis, both of which were attributed to Graves’ disease.

## Summary figure

**Figure:**
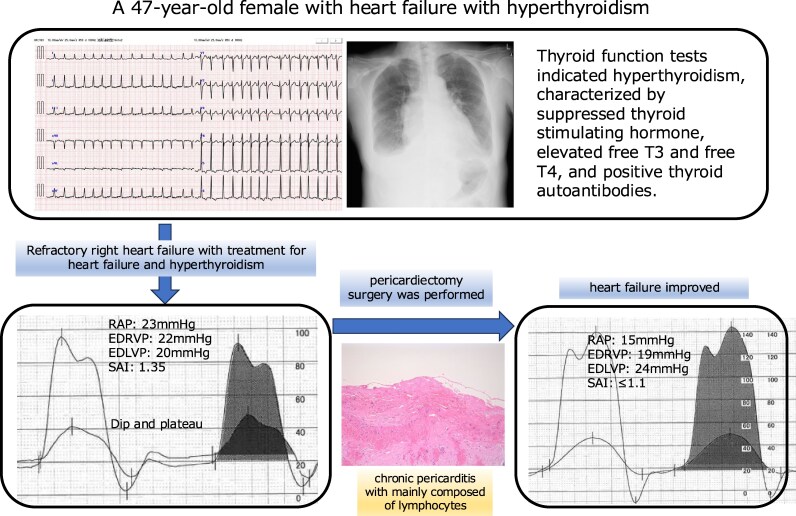
RAP, right atrial pressure; EDRVP, end-diastolic right ventricular pressure; EDLVP, end-diastolic left ventricular pressure; SAI, systolic area index.

## Case presentation

A 47-year-old woman with no past medical, surgical, or radiotherapy history was referred to our institution for progressive dyspnoea. Three years ago, she was diagnosed with tachycardia and had been experiencing exertional dyspnoea over the preceding 2 years. Her symptoms worsened acutely in the week before presentation. She had a history of smoking but did not take regular medications or have relevant family history. She presented with dyspnoea and orthopnoea and was admitted to our hospital with congestive heart failure. On physical examination, the patient had tachycardia and tachypnoea, with a heart rate of 155 beats/min, blood pressure of 129/86 mmHg, and respiratory rate of 24 breaths/min (*Table 1*). Body temperature was elevated to 38°C, whereas oxygen saturation was maintained at 98% on room air. Neck examination revealed a diffusely enlarged thyroid gland without tenderness and jugular venous distention. Pitting oedema was observed in the lower legs.

**Table 1:** Overview of the physical examination, laboratory, and echocardiographic data of the patient

Physical examination data	
Blood pressure (mmHg)	129/86
Heart rate (beats per minute)	155
Respiratory rate (breaths per minute)	24
O_2_ saturation (%)	98
Temperature (°C)	38
Laboratory data	
WBC (/µL; normal 3300–8600)	7600
Haemoglobin (g/dL; normal 11.6–14.8)	11.9
Albumin (g/dL; normal 4.1–5.1)	3.6
Total bilirubin (mg/dL; normal 0.4–1.5)	2.3
Creatinine (mg/dL; normal 0.46–0.79)	0.38
CPK (U/L; normal 41–153)	109
LDL cholesterol (mg/dL; normal 65–163)	79
HDL cholesterol (mg/dL; normal 48–103)	24
CRP (mg/dL; normal < 0.14 mg/dL)	2.47
NT-pro-BNP (pg/mL; normal < 125 pg/mL)	672
Hs-TnT (ng/mL; normal < 0.016 ng/mL)	0.004
Free T3 (ng/dL; normal 2.24–3.94)	16.0
Free T4 (ng/dL; normal 0.77–1.59)	>8.0
TSH (mIU/L; normal 0.61–4.23)	<0.002
TRAb (IU/L; normal < 2.0 IU/L)	10.8
TPOAb (IU/mL; normal < 3.3 IU/mL)	267
TgAb (IU/mL; normal < 19.3 IU/mL)	30.8
Thyroglobulin (ng/mL; normal < 35.1 ng/mL)	230
Echocardiographic data	
LVEDVI (mL/m^2^)	86
LVESVI (mL/m^2^)	51
LVMI (g/m^2^)	96
LAVI (mL/m^2^)	52
LVEF (%)	42
TRPG (mmHg)	34
DCT (ms)	137
E [early diastolic mitral inflow velocity] (cm/s)	120
E/e′ ratio	11

Physical and laboratory data were measured at admission. Echocardiography data were measured 6 days after admission.

WBC, white blood cell; CPK, creatine phosphokinase; LDL, low-density lipoprotein; HDL, high-density lipoprotein; CRP, C-reactive protein; BNP, brain natriuretic peptide; Hs-TnT, high sensitive troponin T; TSH, thyroid-stimulating hormone; TRAb, thyroid-stimulating hormone receptor antibodies; TPOAb, anti-thyroid peroxidase antibody; TgAb, anti-thyroglobulin antibody; LVEDVI, left ventricular end-diastolic volume index; LVESVI, left ventricular end-systolic volume index; LVMI, left ventricular mass index; LAVI, left atrial volume index; LVEF, left ventricular ejection fraction; TRPG, tricuspid regurgitation pressure gradient; DCT, deceleration time.

**Figure 1:**
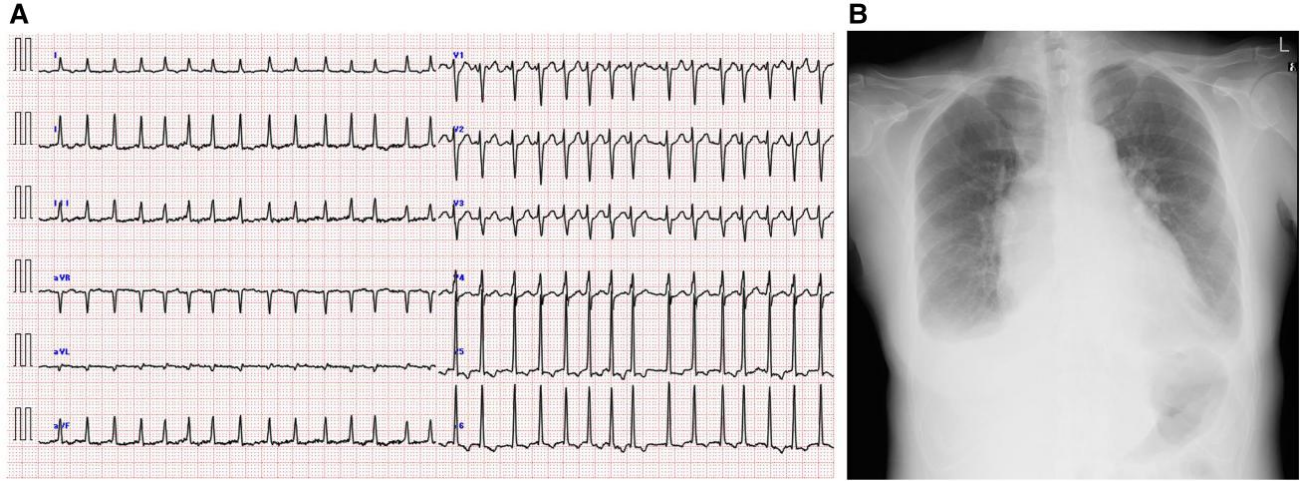
(*A*) Electrocardiogram at the time of admission showing atrial fibrillation with a rapid ventricular response and non-specific ST-segment depression. (*B*) A chest radiograph indicating pulmonary congestion and pleural effusion, with an elevated cardiothoracic ratio.

Electrocardiography revealed atrial fibrillation with a rapid ventricular response and non-specific ST-segment depression (*Figure 1A*). Chest radiography revealed pulmonary congestion and pleural effusion (*Figure 1B*). Mild hypoalbuminaemia, along with an elevated total bilirubin and C-reactive protein levels, was observed. Cardiac biomarkers showed a mildly elevated *n*-terminal pro-brain natriuretic peptide level. Thyroid function tests indicated hyperthyroidism, characterized by suppressed thyroid-stimulating hormone levels, elevated free T3 and T4 levels, and positivity for thyroid autoantibodies. The presence of thyroid-stimulating hormone receptor antibodies supported the diagnosis of Graves’ disease (*Table 1*). Based on these findings, the patient was diagnosed with acute congestive heart failure. The aetiology of heart failure was thought to be catecholamine toxicity and tachycardia with atrial fibrillation associated with Graves’ disease.

**Figure 2:**
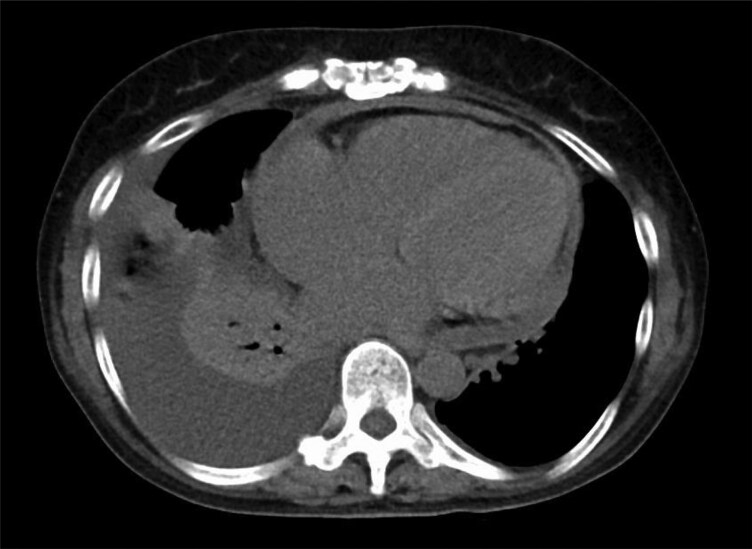
A plain computed tomography scan showing thickening of the pericardium and a small amount of pericardial effusion.

**Figure 3:**
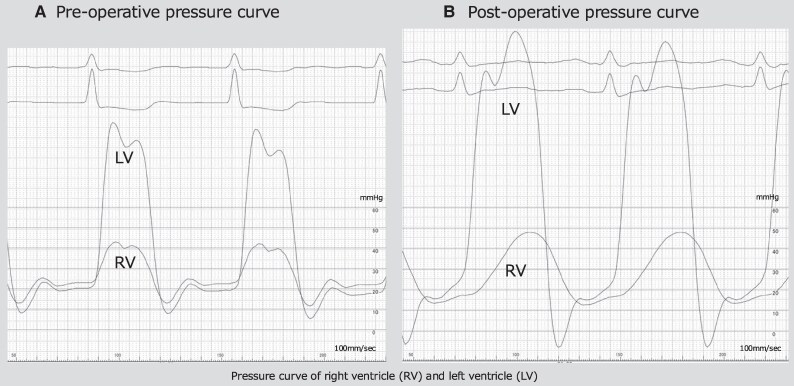
(*A*) The pressure curve of the right ventricle showing a dip and plateau pattern. The end-diastolic pressure of the left and right ventricles was almost equal, at 20 and 22 mmHg, respectively. (*B*) After surgery, the dip and plateau pattern has disappeared in the right ventricular pressure curve on right heart catheterization, with resolved equalization of the end-diastolic pressure of the left and right ventricles.

Continuous intravenous landiolol, diuretics, and thiamazole (30 mg/day) were initiated on the day of admission. After treatment, the thyroid hormone levels normalized, heart rate decreased, and radiography showed improved pulmonary congestion. However, the pleural effusion gradually worsened. Despite increasing the diuretic dose, right-sided heart failure did not improve. On the 11th day of hospitalization, thoracentesis was performed to drain pleural fluid. Pleural effusion was transudative with no abnormalities on bacterial culture or pathological examination. Although the patient received appropriate medical management for heart failure, the clinical signs of right-sided heart failure continued to worsen. Because her condition did not respond well after conventional management, alternative aetiologies were considered. Echocardiography revealed left atrial enlargement, a reduced left ventricular ejection fraction, and pericardial effusion. Echocardiography showed a short deceleration time and elevated early diastolic mitral inflow velocity, suggesting mildly elevated left ventricular filling pressure and left ventricular diastolic dysfunction (*Table 1*). Computed tomography revealed pericardial thickening and effusion (*Figure 2*). Based on these findings, constrictive pericarditis was considered a potential diagnosis. We decided to perform haemodynamic evaluation through catheterization. Left heart catheterization revealed no significant stenosis on coronary angiography. Right heart catheterization revealed an elevated mean right atrial pressure of 23 mmHg and a pulmonary artery wedge pressure of 47 mmHg. The pressure curve of the right ventricle showed a dip and plateau pattern. The end-diastolic pressure of the left and right ventricles was almost equal, at 20 and 22 mmHg, respectively (*Figure 3A*). The systolic area index was 1.35, exceeding the diagnostic threshold of 1.1 for constrictive pericarditis. These findings led to a diagnosis of constrictive pericarditis. Invasive treatment was deemed necessary, and the patient ultimately underwent pericardiectomy.

**Figure 4:**
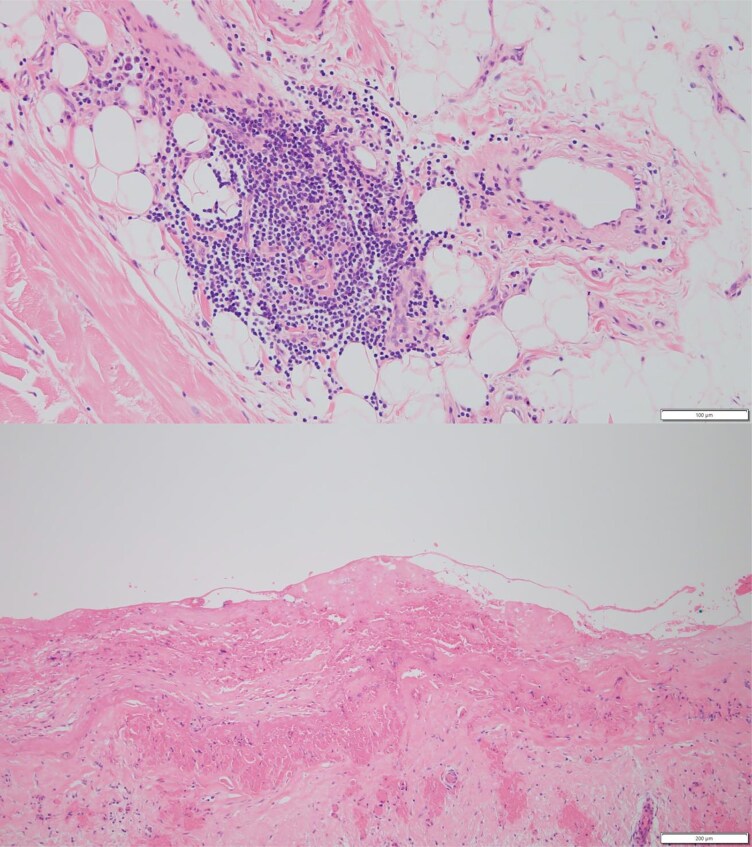
Histological sections of the pericardium showing lymphocytic infiltration forming clusters within the pericardial adipose tissue, consistent with chronic inflammation (H&E, ×100 and ×200).

After surgery, there was a marked improvement in the signs and symptoms of heart failure, allowing a reduction in diuretic dose. In addition, postoperative right heart catheterization confirmed the absence of the dip and plateau in the right ventricular pressure curves, with the systolic area index decreasing to below 1.1 (*Figure 3B*). Histopathological examination of the resected pericardium revealed chronic inflammatory infiltrates, predominantly composed of lymphocytes, particularly within the adjacent adipose tissue. These findings were consistent with chronic pericarditis (*Figure 4*). Over 2 years have passed since the surgery, and no heart failure events have been observed.

## Discussion

We encountered a patient with heart failure, constrictive pericarditis, and Graves’ disease. There have been several reports of an association between thyroid disease and epicardial disease. In particular, there are some reports describing the relationship between acute pericarditis and hyperthyroidism.^1,4–6^ However, no cases of constrictive pericarditis complicated with hyperthyroidism have been reported. In patients with constrictive pericarditis, the inflammatory process typically progresses over time, ultimately resulting in the development of constrictive pericarditis. The patient in the current report did not have any inflammatory diseases known to cause constrictive pericarditis, such as rheumatic diseases, cancer, or infectious diseases. Moreover, the patient did not have any risk factors for constrictive pericarditis such as prior cardiac operations or radiation therapy. Although it is difficult to prove a direct causal relationship between Graves’ disease and constrictive pericarditis in the current study, thyroid disease may have caused pericardial inflammation. Several mechanisms have been proposed to cause pericarditis.^3^ One possible mechanism is that thyrotoxicosis directly induces immune-mediated inflammation. It has also been suggested that systemic viral infections, particularly Epstein-Barr virus infection, may trigger both thyroiditis and pericarditis simultaneously.^1^ The prognosis of constrictive pericarditis varies depending on the underlying cause and timing of treatment. If diagnosed early and treated with anti-inflammatory agents, reversible cases may achieve complete recovery. In the present case, pericarditis was not recent, as evidenced by pericardial thickening and pathological findings consistent with chronic inflammation. Surgical pericardiectomy is often required in patients with chronic fibrosis or calcification. Because the disease had already progressed at the time of diagnosis, surgical intervention was necessary. Pericardiectomy is the recommended treatment for constrictive pericarditis.^7^ Patients with constrictive pericarditis have no specific laboratory or imaging findings, which makes diagnosis difficult. Chest computed tomography revealed mild pericardial thickening, but no calcification. Although echocardiography revealed left atrial enlargement, pericardial effusion, and diastolic dysfunction, these findings alone were insufficient to strongly suspect constrictive pericarditis. Because the right-sided heart failure was refractory to treatment, we performed haemodynamic evaluation through catheterization, which resulted in a diagnosis of constrictive pericarditis. Patients with early surgical intervention after the onset of symptoms have been reported to have better outcomes; therefore, patients with refractory right-sided heart failure should be evaluated with catheterization to assess the possibility of constrictive pericarditis.

When encountering a patient with treatment-resistant right-sided heart failure, it is important to consider the possibility of constrictive pericarditis and evaluate haemodynamics through cardiac catheterization, as appropriate.

## Data Availability

Data supporting the findings of this study are available through the corresponding author upon reasonable request. The data are not publicly available because of privacy and ethical restrictions.

## Lead author biography

Yuki Nakata earned his medical degree from Yamaguchi University, Japan, in 2018. He is currently a general cardiologist and cardiovascular medicine fellow at Tokyo Women’s Medical University Adachi Medical Center. His professional interests focus on interventional cardiology, structural heart disease, and clinical cardiology.

## Notes

**Consent:** Consent was obtained from the patient, and the statement was documented and stored. The authors confirm that written consent for submission and publication of this case report including images and associated text has been obtained the patient, in line with COPE guidance.

## Notes

**Funding:** No financial support was provided for this case.
